# Supplementary material for: Role and mechanism of NCAPD3 in promoting malignant behaviors in gastric cancer
Source: Front Pharmacol. 2024 Apr 22;15:1341039. doi: 10.3389/fphar.2024.1341039 (PMC11070777; doi:10.3389/fphar.2024.1341039)
Supplement: Supplementary file 11 [file DataSheet2.ZIP › GSEA/Oncogenic signature/my_analysis.Gsea.1599461962066/RB_DN.V1_DN.html]

Details for gene set RB\_DN.V1\_DN[GSEA]

|  || Dataset | filtered\_dataset.sample\_info.cls#WT\_versus\_NCAPD3\_MUT |
| Phenotype | sample\_info.cls#WT\_versus\_NCAPD3\_MUT |
| Upregulated in class | NCAPD3\_MUT |
| GeneSet | RB\_DN.V1\_DN |
| Enrichment Score (ES) | -0.5429833 |
| Normalized Enrichment Score (NES) | -2.545265 |
| Nominal p-value | 0.0 |
| FDR q-value | 0.0 |
| FWER p-Value | 0.0 |
Table: GSEA Results Summary

  

Fig 1: Enrichment plot: RB\_DN.V1\_DN      
 Profile of the Running ES Score & Positions of GeneSet Members on the Rank Ordered List

  

| SYMBOL | TITLE | RANK IN GENE LIST | RANK METRIC SCORE | RUNNING ES | CORE ENRICHMENT || 1 | 960 | CD44 | 339 | 0.573 | -0.1967 | No |
| 2 | 51112 | TRAPPC12 | 504 | 0.471 | -0.2759 | No |
| 3 | 3309 | HSPA5 | 876 | -0.288 | -0.5192 | Yes |
| 4 | 2150 | F2RL1 | 887 | -0.304 | -0.5013 | Yes |
| 5 | 8140 | SLC7A5 | 890 | -0.305 | -0.4775 | Yes |
| 6 | 79094 | CHAC1 | 949 | -0.367 | -0.4889 | Yes |
| 7 | 7277 | TUBA4A | 997 | -0.404 | -0.4894 | Yes |
| 8 | 91663 | MYADM | 1004 | -0.407 | -0.4601 | Yes |
| 9 | 3914 | LAMB3 | 1015 | -0.414 | -0.4331 | Yes |
| 10 | 3918 | LAMC2 | 1088 | -0.466 | -0.4464 | Yes |
| 11 | 857 | CAV1 | 1098 | -0.475 | -0.4136 | Yes |
| 12 | 8727 | CTNNAL1 | 1106 | -0.482 | -0.3789 | Yes |
| 13 | 2706 | GJB2 | 1115 | -0.490 | -0.3441 | Yes |
| 14 | 54541 | DDIT4 | 1133 | -0.502 | -0.3149 | Yes |
| 15 | 171177 | RHOV | 1157 | -0.521 | -0.2884 | Yes |
| 16 | 22809 | ATF5 | 1246 | -0.624 | -0.3002 | Yes |
| 17 | 4739 | NEDD9 | 1322 | -0.741 | -0.2931 | Yes |
| 18 | 90865 | IL33 | 1325 | -0.745 | -0.2329 | Yes |
| 19 | 1649 | DDIT3 | 1341 | -0.779 | -0.1793 | Yes |
| 20 | 25819 | NOCT | 1374 | -0.891 | -0.1288 | Yes |
| 21 | 10397 | NDRG1 | 1380 | -0.925 | -0.0560 | Yes |
| 22 | 64764 | CREB3L2 | 1382 | -0.930 | 0.0202 | Yes |
Table: GSEA details [plain text format]

  

Fig 2: RB\_DN.V1\_DN      
 Blue-Pink O' Gram in the Space of the Analyzed GeneSet

  

Fig 3: RB\_DN.V1\_DN: Random ES distribution      
 Gene set null distribution of ES for **RB\_DN.V1\_DN**
